# A critical review of literature on health financing reforms in Uganda – progress, challenges and opportunities for achieving UHC

**DOI:** 10.4314/ahs.v23i1.78

**Published:** 2023-03

**Authors:** Walter Denis Odoch, Flavia Senkubuge, Ann Bosibori Masese, Charles Hongoro

**Affiliations:** 1 School of Health Systems and Public Health (SHSPH), Faculty of Health Sciences, University of Pretoria, Pretoria 0028, Gauteng Province, South Africa; 2 Afya Research and Development Institute, P.O. Box 21743, Plot 2703, Block 208, Bombo Rd, Kampala, Uganda; 3 East, Central and Southern Africa Health Community P.O. Box 1009, Arusha Tanzania; 4 Centre for Health Solutions Kenya; 5 Developmental, Capable and Ethical State (DCE) Division, Human Sciences Research Council of South Africa Private Bag X41, Pretoria, 0001, South Africa

**Keywords:** Uganda, Universal health coverage, health financing

## Abstract

**Background:**

Universal health coverage (UHC) is one of the sustainable development goals (SDG) targets. Progress towards UHC necessitates health financing reforms in many countries. Uganda has had reforms in its health financing, however, there has been no examination of how the reforms align with the principles of financing for UHC.

**Objective:**

This review examines how health financing reforms in Uganda align with UHC principles and contribute to ongoing discussions on financing UHC.

**Methods:**

We conducted a critical review of literature and utilized thematic framework for analysis. Results are presented narratively. The analysis focused on health financing during four health sector strategic plan (HSSP) periods.

**Results:**

In HSSP I, the focus of health financing was on equity, while in HSSP II the focus was on mobilizing more funding. In HSSP III & IV the focus was on financial risk protection and UHC. The changes in focus in health financing objectives have been informed by low per capita expenditures, global level discussions on SDGs and UHC, and the ongoing health financing reform discussions. User fees was abolished in 2001, sector-wide approach was implemented during HSSP I&II, and pilots with results-based financing have occurred. These financing initiatives have not led to significant improvements in financial risk protection as indicated by the high out-of-pocket payments.

**Conclusion:**

Health financing policy intentions were aligned with WHO guidance on reforms towards UHC, however actual outputs and outcomes in terms of improvement in health financing functions and financial risk protections remain far from the intentions.

## Background

In recent years, universal health coverage (UHC), advanced by the World Health Organization (WHO) has gained momentum and dominates global and national discourses. In 2005, the World Health Assembly (WHA) passed resolution WHA58.33 urging member states on UHC and health financing^1^. The United Nations (UN) member states adopted sustainable development goals (SDGs) as the international development blueprint with achieving UHC as one of the targets under goal 3^2^. The SDG target 3.8 is to achieve UHC, including financial risk protection and access to quality essential health care services and access to safe, effective, quality, and affordable essential medicines and vaccines for all^2^–^3^. In 2019, at the United Nations High Level Political Forum meeting in Tokyo Japan, UN member states recommitted to the achievement of UHC^4^.

Universal health coverage means ensuring access to health services for all without financial hardship 5. Therefore, an effective, efficient and equitable health financing system is critical and essential for the achievement of the UHC target under the SDG declaration^6^–^8^. Health financing is the process by which revenues are collected from various sources, accumulated in fund pools and allocated for specific health interventions provided by various healthcare providers to achieve health system goals^9^. The collection of revenue, pooling and accumulation of revenue and purchasing of health services form the three health financing functions.

Health financing influences progress on the three UHC goals of equity in the use of health services, quality of care and financial risk protection through effects on UHC intermediary objectives of transparency and accountability, efficiency and equity in resource distribution^6^,^10^. The movement towards UHC requires health system financing reforms in many countries^6^. Reforms for financing UHC encompass rearrangement in revenue raising, pooling of funds and risks, purchasing and benefit design that aims at improving one or several objectives and goals of health (financing) system, usually measured at the population or system level^11^–^12^. Health financing reforms that facilitate movement towards UHC share certain characteristics, even though policy and programmatic approaches may vary by country 6,13.

There are reports on health financing reforms in Uganda such as those that describe the introduction and later abolition of user fees, implementation of sector wide approach (SWAp) and the proposed national health insurance scheme (NHIS)^14^–^31^. However, there has been no study examining health financing reforms in Uganda in terms of changes in policy intentions (health financing policy objectives), outputs (organization and management of financing functions) and their linkage to outcome (level of financial risk protection) over time, as well as how the reforms have been aligned to WHO principles of reforms that advance UHC.

The current study identifies key characteristics, outputs and outcomes of health financing reform processes in Uganda between 2000 and 2020 and shows how they have been aligned (or not) to the aspirations for achieving UHC. These findings contribute to ongoing discussions on the national health insurance fund (NHIF) and future health financing strategy development. Findings of this review may also be useful to stakeholders from similar contexts who are in the process of reforming health systems financing towards UHC.

## Methods

### Study design and approach

In exploring key features of health financing reforms in Uganda, we used a thematic synthesis approach to the critical review of literature. Thematic synthesis is a qualitative approach that involves selecting, recording and categorizing key issues into themes^32^. For each article, the process involved familiarization with information, identification, recording, categorization^32^. We relied on the use of words, texts and figures to summarize and explain findings on health financing reforms in Uganda between 2000 and 2020.

We reviewed publicly available grey literature and peer-reviewed publications that contained information on health financing in Uganda. These included government development plans, strategies and policies relevant to health financing and reports of other organizations discussing health financing development in Uganda. We also searched electronic databases including Medline (Ovid), PubMed, EBSCO (Medline and CINAHL), Web of Science and Scopus. We used Boolean operator ‘OR’ to combine various conceptual terms of “health financing” and subsequently used Boolean operator ‘AND’ to combine the results of health financing search with “Uganda” (see supplement 1 for search string on PubMed as an example). We also screened reference lists of included studies. The inclusion criteria were: - the document had information on health financing or health financing reform in Uganda, published between 2000 and 2020, and in English. The year 2000 was chosen as a baseline because discussions on UHC gained momentum in early 2000 culminating in the WHA resolution on UHC and health financing in 2005. The exclusion criteria: - the document only had a mention of health financing but did not further describe health financing reforms in Uganda. Non-English documents were also excluded as the translation processes would have required additional resources that the authors did not have. It was also likely that the available English documents would provide adequate data. All eligible documents were exported to EndNote X9^33^ where duplicates were removed. The documents were then exported to NVIVO for analysis.

### Analytical framework

Our analytical approach was informed by McIntyre and Kutzin's^10^ framework that illustrates the relationship between health financing and UHC goals and Kutzin's framework for analysing health financing systems^34^. McIntyre and Kutzin's framework indicate that health financing influences progress toward UHC goals via UHC intermediate objectives of equity in resource distribution, efficiency and transparency and accountability. The UHC goals are equity in the use of services, quality of care and financial risk protection^10^. Kutzin's framework is based on three pillars which include a set of policy objectives that provide the direction in which reforms push the system, functions and policies of the health financing system, and contextual factors^34^. Financial risk protection and equity in the burden of funding the system are generic health financing system objectives (they are also amongst generic health system goals). While transparency and accountability, promoting quality, and efficiency are intermediate health financing objectives^34^.

Therefore, in line with the aim of this study, the following themes were used in the analysis: - Health financing policy statements in the MOH policy documents as policy objectives or intentions (theme 1); how health financing functions are organized and managed to indicate the outputs (theme 2); and level of financial risk protection to indicate the outcomes of the health financing reforms (theme 3). The three themes are related by the fact health financing functions are organized and managed as a process for achieving the health policy objectives (policy intentions) and the outcome of which can be demonstrated by the level of financial risk protection among other indicators (theme 3).

We analysed changes in Uganda's health financing system over four health sector strategic plan periods: July 2000 - June 2005 (Health Sector Strategic Plan I (HSSP I)^35^, July 2005 – June 2010 (Health Sector Strategic Plan II (HSSP II)^36^, July 2010 – June 2015 (Health Sector Strategic & Investment Plan (HSSIP)^37^, and July 2015 – June 2020 (Health Sector Development Plan (HSDP)^37^. These were used as timeframes in the analysis. The HSSIP and HSDP are hereafter referred to as HSSP III and HSSP IV.

Under theme 1, we analysed how health financing policy objectives were stated between HSSP I and HSSP IV, noting areas of relative emphasis and the likely reasons for the changes. In theme 2, on health financing functions, organization and management, we focused on a set of variables under each of the sub-functions and policy on benefits and only used a few health system financing indicators to illustrative the changes over the 4 strategic plan periods. It is not the intention of the current study to delve into all or many of the indicators used in assessing health system financing performance, but to use a few to shed light on the changes that have happened in the various aspects of health financing system over the four strategic plan periods. Under theme 2, in the revenue raising the variables included the source of funds, collection and allocation, and the level of funding. In the pooling function, the variables examined included the pooling agencies, approach to pooling and cross-subsidization. Under purchasing, variables were purchasing organizations and purchasing mechanisms. On the policy on the benefits package, we looked at how it has been defined and financed. The variables were adopted from the WHO guiding principles for health financing reforms that support the achievement of UHC^38^.

In analysing changes in financial risk protection (theme 3) over the four strategic plan periods, we analysed the trend in out-of-pocket (OOP) expenditure over the timeframe. We anticipated paucity in getting data on impoverishing or catastrophic expenditure. However, in low- and middle-income countries OOP expenditure is considered a good proxy for financial risk protection^39^.

### Data extraction

From each of the documents included for analysis we extracted data based on the following thematic areas: - health financing policy objectives, management and organization of health financing functions; and financial risk protection. Quantitative data on selected health financing indicators including the level of financial risk protection are presented as tables in the following section.

## Results and Discussion

**Figure 1:**
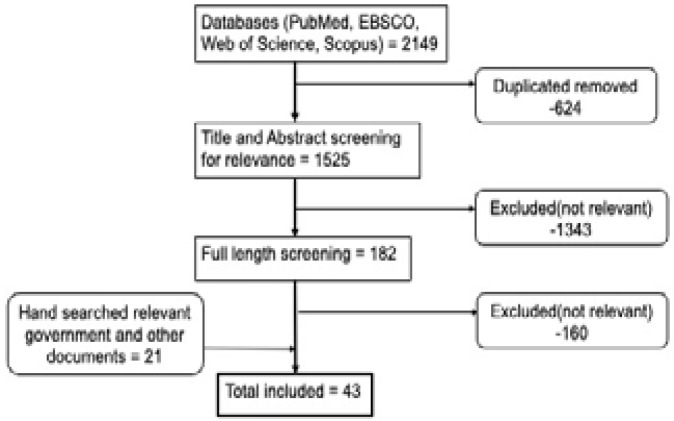
Flow diagram of document selection procedure and results

We identified 43 documents for the review (see fig. 1 and supplement 1). Of these, twenty-two (22) were journal published articles while twenty-one (21) were the government of Uganda, and other institutions' documents that met our inclusion criteria. We present our findings and discussion on the following thematic areas: Health financing policy objectives, health financing system organization, and financial risk protection. In discussing findings, we highlight how changes in health financing were aligned or otherwise to the WHO principles of reforms geared toward UHC.

### Health financing policy objectives

The health financing policy intentions as reflected in policy objective statements have varied in relative emphasis over the four strategic plan periods. In HSSP I the policy emphasized equity and efficiency in resource mobilization, allocation and utilization^35^. The focus of the health financing policy objective during HSSP II was on raising sufficient financial resources for the health sector^36^. During HSSP III and IV, the health financing policy objectives focused on financial risk protection^37^,^40^–^41^. The HSSP III emphasized on ensuring financial risk protection for poor households while HSSP IV envisioned the health financing system attaining UHC through availing required resources for delivery of the essential package of health services^37^,^40^.

Although Uganda's health financing policy objectives have varied in areas of relative emphasis over the four strategic plan periods, they all fall within the broad generic health financing objectives described by Kutzin et al^34^. These include promotion of universal protection against financial risk, equitable distribution of the burden of funding the health system, promotion of equitable use and provision of services relative to need, improving transparency and accountability, and improving efficiency and promotion of quality^34^.

The WHO notes that the relative emphasis with which each country places on a particular generic health financing policy objective varies and may be influenced by specific contextual situations^34^,^42^. In Uganda, the shift in emphasis to resource mobilization as the main health financing policy focus during HSSP II from HSSP I of equity and efficiency was likely occasioned by the low average per capita health expenditure realized during HSSP I. The MOH designed benefits package, the Uganda National Minimum Health Care Package (NMHCP) was costed at $28 per capita for HSSP I, however, the expenditure within that period ranged between $5 and $10 per capita^22^,^24^,^36^. This could have influenced the change of health financing policy focus during HSSP II to mobilization of funding. Financial risk protection and achieving UHC were areas focus of health financing policy objectives during HSSP III and HSSP IV periods. This is likely due to the global level discussions on SDGs and UHC at the time, and the ongoing consultations on NHIF.

### Health financing system organization Revenue raising

The major sources of revenue for health financing throughout the 4 strategic plan periods were: - general government revenue (taxes, concessional loans, grants); private sources (households, private firms, local non-governmental organizations (NGOs); and development partners, donors, global health initiatives (GHI), philanthropists and international NGOs^26^–^28^,^31^,^35^–^37^,^40^–^41^,^43^–^51^. Although the sources have remained the same, there has been some variations over the strategic plan periods in terms of their relative contribution to the total health expenditure (THE) (see table 1).

**Table 1:** Sources of funds of health financing in Uganda from FY 2000/01 to FY 2015/16

Funding Sources	Percentage relative contribution from each source over the years							

	2000/01	2006/07	2008/09	2009/10	2012/13	2013/14	2014/15	2015/16
Public	18.2	15	16	15	16.8	17.7	13.8	15.3
Private (households, private firms and local NGOs)	54.4	57	49	48	43.3	41.1	41.4	42.6
Development partners/donors, INGO, GHI	27.4	28	34	37	38.9	41.2	43.4	41.7
Total Health Exp. (billion Uganda shillings)	745	1609	2808	3234	4866	4952	4944	5309

Source of data: National Health Account reports^40^,^46^,^48^

The two major sources of health financing; households through Out-of-pocket (OOP) payments and development partners' contribution suffer inherent weaknesses. Firstly, OOP payment is associated with inequity in access, catastrophic expenditure and impoverishment^22^,^25^,^28^,^39^,^50^,^52^. Secondly, development partners' funding has issues of unpredictability and fragmentation^27^,^51^,^53^–^55^.

In the public sector, the Ministry of Finance, Planning and Economic Development (MFPED) mobilizes revenue and allocates it to different sectors including health according to priorities set by the government^56^–^58^. Health care providers also collect funds from households through payments made at the point of care^40^,^46^,^48^,^50^,^59^. The other agencies that collect funds for financing health care are the Private Health Insurance (PHI) and Community-Based Health Insurance (CBHI) schemes^40^,^46^,^48^–^49^,^60^–^61^.

Government allocation has been predictable and in real terms increased over the years, with budget execution exceeding 80% of planned health budgets^49^. However, the public fund remains far below the level recommended for the provision of essential health care for a Sub-Saharan Africa country and delivery of NMHCP^28^,^48^–^49^ (see table 2).

**Table 2:** Per capita general government expenditure vs estimates required for delivery of NMHCP between FY 2000/01 and FY 2015/16

Financial year	2000/01	2001/02	2005/06	2007/08	2015/16
Per Capita Government Health expenditure	3.1	7.6	9.98	8.2	9.0
Per capita expenditure required to deliver NMHCP during HSSP I, II&IV	28		34		117

Source of Data: National Health Accounts reports and other reports^24^,^43^,^45^–^46^,^49^,^62^

According to WHO, reforms that advance UHC include a move towards a predominant reliance on public/compulsory funding sources, increase in predictability in the level of funding over a period of years and improvement in stability in the flow of public (and external) funds^38^. Apart from the predictable level in government funding, albeit low, and the policy intentions, the revenue raising function has not changed measurably towards contributing to achievement of UHC as the finding on revenue collection function of the strategic plan periods indicate.

### Pooling of revenue

During HSSP I&II, the MOH and development partners implemented SWAp as a mechanism of pooling development partners funding to finance the national health sector strategic plans. Despite showing promise during HSSP I &II, many partners got discouraged and pulled out of the SWAp arrangement due to suspected misuse of funds^61^,^63^. As a result, many resorted to providing off-budget support directly to service providers.

The MOH first included social health insurance (SHI) as an alternative health financing mechanism in HSSP I following studies conducted in the 1990s. However, not much progress was registered until the HSSP III period when an NHIF Bill was drafted following a cabinet directive^64^. During HSSP III&IV there was a back-and-forth in the process of establishing NHIF. However, by the end of FY 2019/2020, the NHIF law was yet to be enacted. The slow progress has been attributed to challenges including lack of consensus among the reform drivers at MOH; concerns about costs, administrative set-up, institutional capacity for purchasing and regulation of pricing of services in the public and private sectors; and political economy factors including push-back by key political constituencies such as employer groups, implications on the cost of industrial productions and regional competitiveness, among others^41^,^64^–^65^. Key stakeholders driving the process of establishing the NHIF may consider proposing other options, especially in terms of sources of financing. For example, as a starting point, reorganize and use existing sources of funding without the need for the proposed requirements of additional sources of funds from employees and employers as is the case with the United Kingdom National Health Service^66^. This may require some reforms in organization of the health system, however it may be more acceptable, as it can be fronted as an efficiency improvement intervention since there will be no significant extra funding for its establishment.

The only large and predictable prepaid fund remains the public funds allocated to MOH and those mobilized from external sources and managed by MOH which accounts for only about 18% of THE

Non-pooled funds contribute the largest proportion to THE; accounting for between 37% and 51% of THE over the four strategic plan periods^40^–^41^,^48^. Therefore, the picture of pooling in Uganda departs from principles of reforms in health financing for UHC where there should be progressive reliance on pooled public funds, progressive reduction in the proportion of OOP payment to THE, and reduced fragmentation^6^,^34^.

### Purchasing

The purchasing entities of health services were the same throughout the four strategic plan periods^27^,^35^–^37^,^47^,^54^. They included MOH and local government authorities, households, NGOs and private health insurers. In terms of total purchase, the government and NGOs purchased about 25% of health services each, while households purchased about 50%, and PHI and CBHI scheme purchased less than 1% during the strategic plan periods^27^,^40^,^46^,^48^.

Payment mechanism in the public sector has remained line-item input-based through government budgetary allocation during all 4 strategic plan periods. Government allocation to public and private-not-for-profit (PNFP) health facilities is based on a formula that takes into account historical costs, geographical location, and epidemiological and demographic characteristics^27^,^59^,^67^. The government conditional grants to PNFP are in return for access to health care by the catchment area population at subsidized cost^23^,^44^,^67^. Private Health Insurance Schemes and households pay private health providers for selected services in public facilities based on a fee-for-service arrangement. Before its abolition in 2001, households paid user-fee in all public health facilities^24^,^52^,^61^. Abolition of user fees in 2001 was due to a combination of factors such as low contribution to health financing (around 5% of total health facility funding^52^, high administration costs, limited access to care by the poor and need to lower OOP expenditure^36^. However, this has not lowered OOP payments which remains the main approach to purchasing health care^24^,^41^,^52^.

During the HSSP III period, a national framework for results-based financing (RBF) and its implementation manual were developed based on results from pilot RBF projects^41^. The pilot projects were scaled-up during HSSP IV period^49^,^68^. However, RBF does not form the main mechanism for purchasing health care, it rather targets improvement in the demand and supply side interventions mainly for maternal and child health services^41^. In addition, the government introduced an output-oriented budgeting approach; program-based budgeting (PBB) from the financial year 2016/17. This led to progressive incorporation of performance measures into the public financial management system^41^,^49^.

The NHIS is being developed as an output-based purchaser to facilitate provider-purchaser split in the public sector to encourage strategic purchasing^41^,^65^. However, as noted above, after a two-decade process, efforts to establish a NHIS as an output-based purchaser has stalled over several concerns aforementioned^28^,^41^,^58^,^64^.

Therefore, apart from the pilots with RBF and some PBB, the traditional formula input-based approach quarterly payment to public agencies and conditional grants to PNFP remains the main mode of health care purchasing in the public sector. The purchasing approach during the 4 strategic plan periods is counter to the WHO recommendation of an effective provider-purchaser split as one of the mechanisms that facilitate strategic purchasing and enable a move towards UHC^6^,^34^.

### Policy on the benefits package

The NMHCP remains the package entitled to the population. It describes the type of health services offered at each level of the health system. This package should be free of charge at the point of care in public health facilities (except in the private wings)^35^,^37^,^47^,^69^, and is subsidized in PNFP health facilities. However, availability of this package is not guaranteed as it depends on the availability of funding allocated yearly to MOH and this has always fallen short of levels required over the four strategic plan periods (see table 2)^36^,^45^,^49^,^56^. The PHI and CBHI schemes offer various categories of insurance premiums to their members, each with defined sets of services and prices. The WHO recommends transparency and accountability in the delivery of the benefits package with clearly defined legal entitlements to benefits and transparent rationing mechanisms^65^.

### Financial risk protection

The OOP expenditure ranged between 37% and 51% of total health expenditure (THE) over the four strategic plan periods (table 3).

**Table 3:** OOP expenditure as a percentage of total health expenditure between FY2000/01 and FY2015/16

Financial Year	2000/01	2006/07	2008/09	2009/10	2012/13	2014/15	2015/16
OOP as % THE	41	51	40	40	41	33	37

Source of data: National Health Account reports^40^,^46^,^48^

As a measure of financial risk protection, it has been observed that household catastrophic health spending and impoverishment remain low in countries where OOP is less than 20% THE^70^. Among the government intentions of abolishing user-fee in 2001 was to increase access to care especially for the poor and reduce unwanted effects of OOP payments. However, even with the increasing donor flow for health, government subsidies to PNFPs, OOP payment remains the largest form of payment for health care in Uganda^22^,^24^–^25^,^28^,^52^,^59^. Therefore, the policy reforms in terms of the abolition of user fee, subsides at PNFP health facilities and harnessing development partners funding including through the SWAP and RBF has not improved financial risk protection.

## Conclusion

The general structure of how the health system financing is organized in Uganda has not changed appreciably over the last twenty years, despite variation in health financing policy objectives over the four strategic plan periods. The composition of the three main health financing sources; the public funds, the development partners, and households maintained the same trend. Household payment via OOP remained the main source of health financing. Nevertheless, some features of reforms in health system financing can be discerned. These include the abolition of user fees in public health facilities, development of NMHCP as the benefits package, establishment of SWAp, movement towards performance-based financing exhibited by RBF pilot projects and PBB.

Uganda has had good policy intentions as demonstrated by the health financing policy statements during the 4 strategic plan periods that were aligned to the WHO health financing reform principles that advance UHC. However, reforming health financing organizations and management such as the abolition of user fees in public health facilities, SWAp, RBF, PBB and government subsidies to PNFP health facilities have not led to desired health financing system outcome in terms of improved financial risk protection (the OOP remained very high). The low progress to achieving health system goals may be attributed to political, technical and economic challenges often associated with designing, developing and implementation of policy reforms. This paper did not examine other health financing systems' objective of equity in the distribution of the burden of funding the health system as an outcome, however, where OOP is high, the system tends to be very inequitable as the sick who are usually the poor also tend to pay more.

Attempts to establish a NHIF and use it as a catalyst towards a comprehensive health system reform has been prolonged for over two decades. There is need for a comprehensive assessment of the bottlenecks as well as consideration of other options for improving pooling, purchasing and accountability. The drivers of the reform could also benefit from a better appreciation of the political economy issues and harness existing high-level commitment on UHC enshrined in the SDGs^2^, the recent high-level meeting of the UNGA where member states recommitted to achieving UHC^4^. In addition, policy lessons from abolition of user-fee and the implementation of SWAp need to be considered during the developments of NHIF and in the improvement of the overall health financing functions.
